# ‘In the end, I’m the one who has to do the job’: Participant experience of a lifestyle intervention for patients with hypertension

**DOI:** 10.1080/02813432.2023.2271042

**Published:** 2023-11-29

**Authors:** Hanna Glock, Beata Borgström Bolmsjö, Veronica Milos Nymberg, Moa Wolff, Susanna Calling

**Affiliations:** Center for Primary Health Care Research, Department of Clinical Sciences Malmö, Lund University, Lund, Sweden

**Keywords:** text messaging, hypertension, lifestyle, primary care, qualitative research, theory of planned behaviour

## Abstract

**Objective:**

Cardiovascular disease can be prevented through lifestyle changes, but such changes are often hard to attain. Text message interventions with lifestyle advice have shown small but promising effects. Our objective was to explore participant experience of a text message lifestyle intervention for patients with hypertension, and implications for future lifestyle interventions.

**Design and subjects:**

Fourteen participants were purposefully selected for telephone interviews after completion of a text message lifestyle intervention. A semi-structured interview guide with open-ended questions was used. Interviews were recorded and transcribed verbatim. The material was analyzed through systematic text condensation as described by Malterud, a data-driven analysis style that includes deductive elements.

**Setting:**

Primary care in three regions in southern Sweden.

**Results:**

The analysis resulted in three themes. ‘Blood pressure and lifestyle, how seriously to take it?’ revealed a remaining need for knowledge regarding to what extent lifestyle affects risk for cardiovascular disease. ‘The text messages – a useful reminder in need of tailoring’ described that the design of the intervention was satisfactory, but suggested improvements through increased individualization. Finally, ‘Water off a duck’s back or a kick in the pants – when does behavior change?’ showed how some participants responded to the push to change while others did not, supplying material for further analysis in a framework of behavioral change theory.

**Conclusion:**

A key to adoption was advice that was applicable in daily life. Timing in relation to a diagnosis of hypertension or other motivational factors, and tailoring to prior knowledge, habits, and limitations could increase effectiveness.

## Key points

- Text message interventions with lifestyle advice for hypertension and cardiovascular disease have shown small effects, but effectiveness may be improved.
- Our qualitative analysis of patient experience revealed a remaining need for increased knowledge regarding blood pressure, lifestyle, and cardiovascular disease.
- Text message advice that was applicable in daily life, or perceived as new, seemed most useful to facilitate lifestyle change.
- Timing in relation to motivational factors and tailoring to individual habits and limitations could increase effectiveness of similar interventions.

## Introduction

High systolic blood pressure is the leading global risk factor for mortality, accounting for almost 11 million (19%) attributable deaths worldwide in 2019, mainly through cardiovascular disease (CVD) [1]. The prevalence of hypertension is highest in low- and middle income countries [2]. It has decreased over time in high-income countries but remains a major risk factor for mortality [1, 2]. Similarly, CVD mortality has dramatically decreased in high-income countries since the 1960s [3]. However, it is still the leading cause of death, and a rise has been indicated in some high-income regions in recent years [3, 4].

Hypertension is primarily treated pharmacologically, though non-pharmacological interventions are also recommended [2]. CVD, in turn, can to some extent be prevented through lifestyle changes. According to a meta-analysis, adopting more than one healthy habit (regarding physical activity, smoking, diet, alcohol consumption, and/or body weight) reduces the risk of CVD by 66% [5]. However, European primary care patients with newly diagnosed hypertension exhibit poor risk factor control [6, 7].

Digital interventions have been put forth as one way to improve prevention [8, 9]. A meta-analysis of randomized controlled trials (RCTs) that employed digital interventions for risk factor modification in patients with CVD showed effects on healthy habits, but not on unhealthy habits or clinical outcomes [10]. However, a meta-analysis specifically regarding text message (SMS) interventions for CVD prevention found small effects on blood pressure and body mass index (BMI), while effects on multiple risk factors remained uncertain [11]. A third, recent meta-analysis found that text messages and other digital interventions for the treatment of hypertension could provide a small but significant reduction in systolic blood pressure [12]. Consequently, text messaging has arisen as a promising mode of delivery for lifestyle advice to patients with hypertension. However, there may be opportunities to increase effectiveness.

There is a relative paucity of research on patient experience of text messages as a lifestyle intervention, especially if the focus is narrowed to patients with hypertension in primary care. Existing studies have indicated that engagement with and personalization of content play a role, but more research has been called for [12–16].

Cognitive behavioral theories can be used to develop and interpret digital interventions for cardiovascular prevention [17]. An often cited model is the Theory of Planned Behavior (TPB), which was developed from preceding theories in 1991 and has since been extensively applied in the study of health behaviors [17–20]. The Reasoned Action Approach (RAA) is a further development that is increasingly used [19–21]. In short, the RAA states that three factors with dual aspects strongly predict behavioral intention and, by extension, individual behavior: attitude toward a behavior (experiential/instrumental), perceived norm (injunctive/descriptive), and perceived behavioral control (capacity/autonomy). The three predictors depend on an individual’s underlying beliefs, which in turn depend on several background factors. The transformation of intention into behavior is moderated by actual control (skills, abilities, and environmental factors) [19–21].

In 2018, we conducted a pilot RCT evaluating text messages as an intervention to promote lifestyle changes in individuals with hypertension in Swedish primary care [22]. The pilot study showed the feasibility of the study protocol and favorable trends for blood pressure and other cardiovascular outcome measures. More than 90% of the participants reported that they read all the text messages, and 76% agreed that the text messages served to remind them about healthy habits. Some participants requested more advanced information [22]. The text messages were edited using the feedback, and the pilot study was continued into a full-scale RCT, Primary care USage of Health promoting MEssages (PUSHME, ClinicalTrials.gov identifier: NCT04407962).

In summary, lifestyle changes are of major importance for CVD prevention in patients with hypertension, but more efficient interventions are needed. Text messages have the potential to serve as a simple and useful medium for lifestyle advice. However, there is little research on primary care patients’ experience of interventions such as PUSHME. To understand health behaviors, cognitive behavioral theories have proved useful with the RAA being a relatively recent but well-founded development.

Consequently, to explore participant experience of text messages as a lifestyle intervention for patients with hypertension in primary care, and implications for future lifestyle interventions, we extended the PUSHME RCT with a follow-up interview study using the RAA as a framework.

## Material and methods

### Setting and design

The participants in the interview study were selected from the intervention arm of PUSHME RCT, which was performed in primary care in three regions in southern Sweden. The intervention consisted of one-way text messages with lifestyle advice for patients with hypertension, in addition to treatment as usual. The messages had been developed by the researchers based on Swedish national guidelines and edited using feedback from the pilot trial as well as from an expert group at the Regional Center for Lifestyle Habits. Most messages were text-only, but some included links to educational material (Supplement 1). The messages were delivered four times per week for six months, in random sequence and at random times between 9AM and 7PM. Each week, four areas were covered: physical activity, tobacco (for smokers only), dietary habits, and general cardiovascular health (alcohol, stress, medication, and general information). All messages began with ‘Hi [name]…’. A baseline visit was performed before randomization, and a follow-up control was performed after six months. The primary outcome measure of the RCT was change in blood pressure. Secondary outcomes included changes in other cardiovascular risk factors. Patients also completed a questionnaire based on the Theory of Planned Behavior for separate analysis.

### Recruitment and participants

From September 2021 to December 2022, at the follow-up visit of the RCT, 159 patients were asked whether they would like to participate in a telephone interview regarding their experiences of receiving text messages as a lifestyle intervention. A total of 116 patients agreed, while 43 did not consent to participate. From those who agreed, participants were selected and approached via phone call. Purposive sampling was used to include participants with different characteristics regarding sex, age, geographical location, education, number of years with hypertension, blood pressure levels, reported habits, and Swedish language proficiency. All 14 participants who were approached via phone call agreed to participate.

### Data collection

A semi-structured interview guide with open-ended questions was developed by the first author (HG) based on the objective, characteristics of the intervention, and the RAA. The interview guide was discussed among all authors. After the first two interviews, the guide was evaluated and adjusted (Supplement 2). The interview guide was used as a memory aid to ensure that important topics were covered, but all interviews were adapted to the individual participant [23].

Telephone interviews were conducted by HG, who is a resident physician in general practice. After each interview, HG wrote memos regarding the general impression. The interviews were recorded and transcribed verbatim. The first seven interviews were transcribed by HG, and the last seven interviews by a professional transcriber. Interviews were conducted one to four months (mean three months) after the text message intervention. Mean duration of the interviews was 41 minutes (range 26-58 minutes).

### Data analysis

Pseudonymized transcripts were processed in NVivo software (release 1.7.1, QSR international). The material was analyzed through systematic text condensation as described by Malterud, which is an editing (data-driven) analysis style that also includes deductive (theory-driven) elements [23, 24]. Analysis was performed in parallel with the interviews. Putting theoretical framework and preconceptions aside, the first four interviews were read repeatedly by three of the researchers to find preliminary themes connecting to the objective. The separate lists of preliminary themes were discussed among the researchers to arrive at joint preliminary themes through observer triangulation [23]. The first four interviews were then read by HG to find meaning units. The meaning units were sorted into code groups based on the preliminary themes. Each code group was sorted into subgroups. For each subgroup, the meaning units were transformed into condensates. A quotation was chosen to illustrate each subgroup. The condensates were abstracted into an analytical text, using the code groups and subgroups as a structural framework. At this stage of analysis, the theoretical framework of the RAA was also considered, and the results were reconnected to the research question. The described stepwise sequence of analysis was then reiterated for three to four additional interviews at a time. After a total of 14 interviews and four sequences of analysis, no significant addition to answer the research question was deemed to be gained from further interviews. At all stages of analysis, the material was used to develop and reorganize code groups and subgroups. Separate analytical steps including coding, and the evolving analytical text, were triangulated among all researchers at several time-points [23, 24]. Final categories and overarching themes were also triangulated among all researchers [23–25]. The results were recontextualized through re-reading of the full transcripts [23, 24]. Examples of final coding are shown in Table 1. The Consolidated criteria for reporting qualitative research (COREQ) checklist was used in the preparation of the manuscript [26].

**Table 1.** Examples of final coding.

Meaning unit	Subcategory	Category	Theme
‘The public, so to speak, everyone talks about their blood pressure, but what is blood pressure?’	Thoughts about blood pressure	High blood pressure, is it serious or not?	Blood pressure and lifestyle, how seriously to take it?
‘You think that life can disappear so fast, too.’	Fear of cardiovascular disease		
‘Yes, above all I think it is important to exercise. And I think that can fundamentally affect it for the better.’	Lifestyle affects blood pressure	Lifestyle is important for blood pressure – right?	
‘No, that [lifestyle advice] has not come up when I have seen the doctor or the nurse.’	Lifestyle during health care visits		

### Ethical considerations

The RCT and the interview study were approved by the Swedish Ethical Review Authority (dnr 2019-06361 and dnr 2021-02802). The studies were conducted in accordance with the protocol and applicable regulatory requirements. All patients were informed about the study, both in written form and orally. An informed consent form was signed and collected before enrolment.

## Results

**Figure 1.**
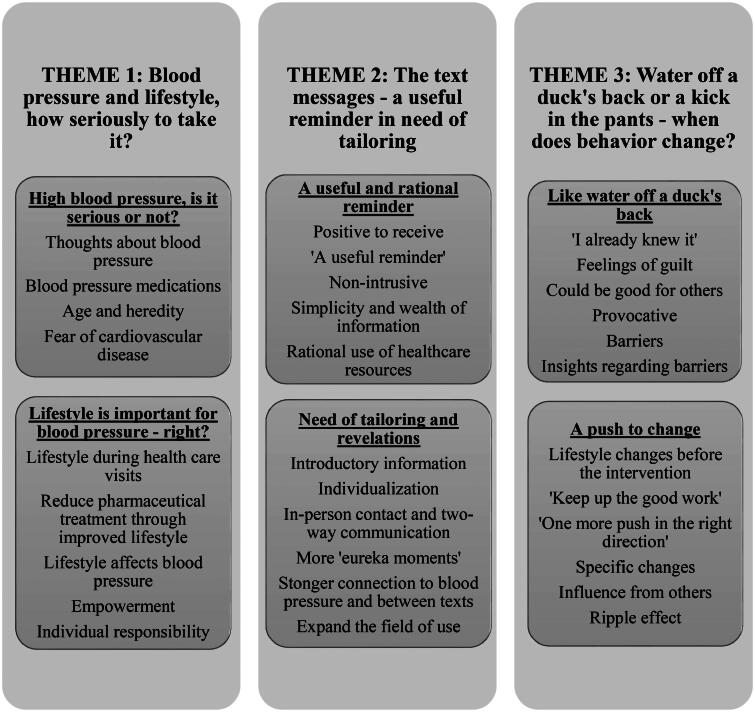
Themes, categories, and subcategories.

Participant baseline characteristics are summarized in Table 2. The interviews provided information on general perceptions of blood pressure and lifestyle, specific experiences of the intervention, and barriers and facilitators to lifestyle change. The analysis resulted in three themes: ‘Blood pressure and lifestyle, how seriously to take it?’, ‘The text messages –a useful reminder in need of tailoring’, and ‘Water off a duck’s back or a kick in the pants – when does behavior change?’. Each theme emerged from two categories and several subcategories (Figure 1). In the following analytical text, themes and categories are signaled as headings and subheadings while subcategories are *italicized*.

**Table 2.** Participant baseline characteristics (N = 14).

Characteristic	n (%)	Mean (SD)	Range
Age (years)		66 (11)	41-82
Sex, male	6 (43)		
University education	8 (57)		
Swedish as a second language	2 (14)		
Alcohol >9 units per week	3 (21)		
Physical activity^a^ <3 h per week	9 (64)		
No exercise^b^	7 (50)		
Hypertension for <2 years	2 (14)		
Hypertension for >10 years	6 (43)		
Systolic blood pressure		144 (13)	124-168
Diastolic blood pressure		88 (11)	73-110

^a^Moderate physical activity such as walking, cycling, gardening (at least 10 minutes at a time).

^b^Vigorous physical activity such as running, aerobics, ball games.

### Theme 1: Blood pressure and lifestyle, how seriously to take it?

#### High blood pressure, is it serious or not?

The respondents’ primary *thoughts about blood pressure* concerned the need for regular checks and normal values. There was uncertainty regarding whether high blood pressure was a risk factor or a disease. *Blood pressure medications* were primarily perceived as a necessity that had to be taken as prescribed. *Age and heredity* were mentioned as two non-modifiable causes of high blood pressure. Some participants expressed *fear of cardiovascular disease*, often in the context of relatives who had become ill or personal cardiovascular events. The degree of anxiety varied greatly.

Yes, yes, the blood pressure, it… Maybe you don’t take it that seriously, when you don’t have so to say any pain from it. (#14)

And then she had a stroke. So you think that, so that you don’t have one yourself. But she had a very high blood pressure. (#5)

#### Lifestyle is important for blood pressure – right?

Some participants described, with appreciation, conversations about *lifestyle during health care visits* for hypertension. Others put forth that lifestyle was not discussed at all or merely superficially during visits for hypertension. The participants were generally positive regarding an increased focus on lifestyle. Several respondents expressed expectations to *reduce pharmaceutical treatment through improved lifestyle*. However, regarding specific questions about risk and treatment, great trust was put in the physician.

The participants all agreed that *lifestyle affects blood pressure*. Diet, physical activity, alcohol, smoking, and stress were mentioned as important factors. However, there was some insecurity regarding to what extent lifestyle can affect blood pressure, where perceptions varied between participants as well as during the same interview.

Yes, above all it’s the alcohol. Then of course heredity can be of some importance. And maybe also what you eat and what kind of lifestyle you have and such. (…) No, above all it’s that. And maybe a little bit the alcohol. (#6)

The respondents almost exclusively reported high *empowerment* and a definitive sense of *individual responsibility* for their lifestyle, even if others could affect them.

In the end it’s that I have to, I’m the one who has to do the job. And to take an interest is really important. (#4)

### Theme 2: The text messages – a useful reminder in need of tailoring

#### A useful and rational reminder

The intervention was generally experienced as agreeable, and as something that could be recommended to others. The text messages were usually perceived as *positive to receive*. It was rather common for participants to share the messages with their closest family.

The participants described the text messages as *‘a useful reminder’* with adequate health advice, and good suggestions and examples. Participants with a professional background in health care also perceived the text messages to be valuable. Duration and frequency were overall perceived as feasible and *non-intrusive*. A few participants would have preferred fewer text messages or a shorter duration. Others missed the messages on the days when they did not receive any.

The respondents did not experience any difficulties in receiving or understanding the text messages. This also applied to participants of an older age and those who were not native Swedish speakers. The *simplicity and wealth of information*, and the possibility to read the messages when convenient, were seen as advantages compared to in-person contacts.

Lifestyle advice via text message was perceived as a *rational use of health care resources*. The participants appreciated receiving the information when they did not need to feel stressed, as during a health care visit, but still from a reliable and well-known source.

You go and sit down on a couch or whatever, you pick up the phone and then you see that you have gotten a text. That’s actually a perfect moment to get the message. (#8)

Sure, you can sit and talk for 10 minutes with a physician or so, but you do not at all bring as much [information] with you. Now, you can read it through twice, if you want to. (#3)

#### Need of tailoring and revelations

There appeared to be some need for more comprehensible *introductory information* regarding the content and purpose of the text message intervention. However, above all, the participants requested more *individualization*. They wanted the lifestyle advice to be modified according to factors such as diseases, age, current lifestyle, interests, and personal circumstances. Some respondents requested the possibility of *in-person contact* with a physician or a nurse, for questions and individualized advice.

Texts are good too, but sometimes, it… it’s a bit too cold. (#11)

A few participants suggested *two-way communication*, i.e., the possibility to reply to the text messages with follow-up questions or suggestions to other participants. Some respondents expressed a need for more memorable advice and new perspectives, in the words of one participant, *more ‘eureka moments’*.

Well, it isn’t anything that’s given me an eureka moment or any kind of… (#9)

To mitigate the impression of ‘general health advice’ a *stronger connection to blood pressure and between texts*, for example through thematization on subjects such as alcohol, was also suggested. Further, there were ideas to *expand the field of use* through repeated interventions, similar interventions for other diseases, or use in public health work. Not all participants appreciated or clicked on the hyper-links that were included in some text messages, and suggestions were made to include videos instead.

### Theme 3: Water off a duck’s back or a kick in the pants – when does behavior change?

#### Like water off a duck’s back

The participants expressed different versions of the statement *‘I already knew it’*, i.e., that they were aware of most or all information that was included in the text messages.

Because often you might think ‘I already knew it’. It isn’t any secrets and news about how you should lead your life and what you should do and so on. Most people already know all of this. (#8)

Some respondents thought that they already led their lives according to the recommendations, and that they did not need to change anything in their lifestyle. Others conveyed that the text messages had not affected their lifestyle because they either simply had not ‘taken it in’ or had deliberately chosen not to follow the advice. Some *feelings of guilt* emerged. The participants described that it was hard to follow all advice regarding diet, physical activity, and alcohol consumption, but that they tried or should. The text messages could therefore cause anxiety. However, it was also put forth that many people were ‘a lot more sloppy’ than oneself, and that the information thus *could be good for others*.

A few participants noted that certain acquaintances would have ‘lashed out’ at some categories of text messages, as they did not take an interest or had a negative attitude. A small number of participants also expressed that some specific advice was not applicable to them, and thereby perceived as annoying or even *provocative*. Other, more tangible *barriers* to adopting the intervention were also expressed in the interviews. The participants wanted to lead a ‘normal’ life and not feel ill. They did not want to abstain from the good things in life. It could be perceived as difficult to find acceptable dietary alternatives. The options for physical activity were also limited by individual preferences and diseases.

The participants expressed some *insights regarding barriers* to implementing lifestyle changes. They then acknowledged that they did not always follow all advice, and that they rejected information about risk connected to lifestyle through ‘some kind of defense mechanism’. A few participants described that they did not implement potential lifestyle changes due to ‘made-up barriers’ or ‘a certain habit’. To affect motivation and break old habits were suggested as keys to lifestyle change.

I didn’t know everything, but a lot of it I already knew, just that I have kind of ignored it, ‘it doesn’t happen to me’ [laughter]. (#13)

#### A push to change

Some participants had implemented *lifestyle changes before the intervention*, for example after a precipitating event such as when they got the diagnosis of hypertension or if they or a relative had gotten a heart attack or some other CVD. Those participants could perceive the text messages as a confirmation, or a reminder to *‘keep up the good work’*. However, more often the text messages were perceived as ‘food for thought’ or ‘o*ne more push in the right direction*’ to actually realize a lifestyle change. Information about diseases or risk factors in temporal proximity to the intervention seemed to work in synergy with the ‘push’ from the text messages, generating a ‘double kick-start’. The text messages could also evoke the feeling that ‘someone is with me’, which increased the motivation to change.

You could say that most of the texts that came I was kind of aware of, but I got a… I got a kick in the pants when they came. (#13)

But it pushed me, because there is always something that you can learn a bit more about. And then you have to decide that you will follow it, that you do it. (#4)

Several participants described that the text messages made it more evident how they should lead their lives, and that their lifestyle habits actually mattered. Some were pushed to implement advice that they had already been thinking about, for example to increase their physical activity levels. For others, there could be an eye-opening effect from suggestions such as abstaining from alcohol or increasing the amount of vegetables they ate. The participants also provided a number of examples of *specific changes* that they had implemented in connection with the intervention. Areas of change included a healthier diet and increased physical activity as well as decreased alcohol consumption and smoking.

I began to do group exercise and I think that has been really good, that I got started with it. Before, I guess I did some things, went out for a walk and such, but maybe not as regularly as I have done later. (#2)

The common denominator was a change of routines and that the participants perceived that the text messages had played a role in this, even if the full reasons for a change were sometimes hard to pinpoint. Realized changes – before or during the intervention – could give rise to a spiral of improved general health, perseverance, and further implementation of lifestyle changes.

But I cut down on everything and began to take care of myself, and then I got a stationary bike that I sit and exercise on a bit at home. (#13)

*Influence from others*, primarily family, was perceived to contribute to lifestyle changes through information and inspiration. In the other direction, the text messages could initiate a *ripple effect* when participants shared information or occasionally brought family or friends with them in their new habits.

## Discussion

### Principal findings

This qualitative analysis of participant experience of lifestyle-promoting text messages for patients with hypertension resulted in three themes. ‘Blood pressure and lifestyle, how seriously to take it?’ revealed a remaining need for increased knowledge regarding hypertension and to what extent lifestyle affects the risk for CVD. ‘The text messages – a useful reminder in need of tailoring’ described that the design of the intervention was satisfactory, agreed with prior research in requesting increased individualization and further suggested more ‘eureka moments’. Finally, ‘Water off a duck’s back or a kick in the pants – when does behavior change?’ provided detailed information on how some participants responded to the push to change while others did not, supplying material for further analysis in a framework of behavioral change theory.

### Findings in a theoretical framework

**Figure 2.**
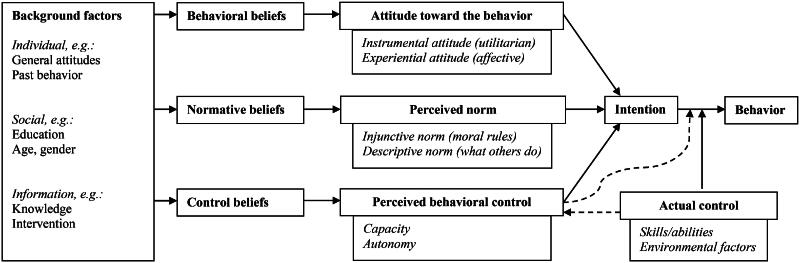
Schematic presentation of the Reasoned Action Approach, adapted from Fishbein & Ajzen [21].

**Figure 3.**
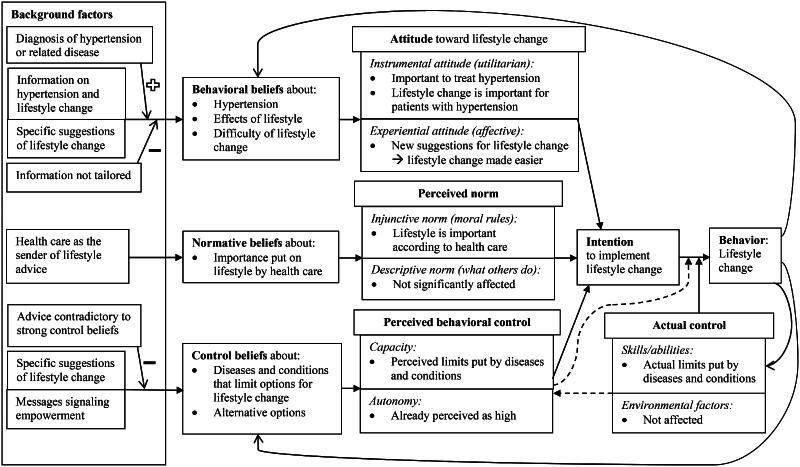
PUSHME interview results in the framework of the Reasoned Action Approach, adapted from Fishbein & Ajzen [21]. Text in bold and italics, and closed arrows, show the framework of the original model. For each block of the model, factors that emerged as important in the analysis of the interviews are spelled out. Open arrows are additions compared to the original model.

The results can be interpreted in the framework of the RAA (Figures 2 and 3) [19–21, 27]. As suggested by Ajzen, the intervention is understood as a background factor [19, 21]. As such, the text messages positively affected behavioral beliefs about the importance of lifestyle change for patients with hypertension, and thereby instrumental (utilitarian) attitude toward lifestyle change. Furthermore, text messages with specific suggestions may have altered beliefs about the difficulty of lifestyle changes and thereby experiential (affective) attitudes. The second predictor of intention, normative beliefs, was likely influenced by the fact that the sender of the text messages was a health care institution. This positively affected the perceived injunctive norm that lifestyle is important according to health care services. Considering the third predictor of intention – perceived behavioral control – the participants already expressed beliefs that centered on individual responsibility for lifestyle changes. The text messages primarily contributed with options for changes that the participant could carry out, thereby countering the perceived limitations of diseases and conditions, and increasing perceived capacity. The three predictors then affected the intention to perform lifestyle change and contributed to the participant realizing a lifestyle change.

As illustrated by the plus sign in Figure 3, the effect of the text messages on behavioral beliefs could be reinforced by events that increased motivation to change, such as a first-time diagnosis of hypertension. Effects were mitigated by the intervention not being tailored to participants’ prior knowledge and habits. Also, advice contradictory to strong control beliefs caused feelings of being provoked, and decreased adoption. Finally, experience of lifestyle change could facilitate further lifestyle change in a positive feedback loop.

In summary, connecting the results of systematic text condensation to the RAA indicates two important factors for the effects of the intervention. The first is the message that lifestyle change is important for patients with hypertension, and strongly recommended by health care providers. The second is suggestions of actual changes that can be implemented by the participants and thus contribute to further lifestyle change. The modifiers added to the theoretical framework contribute with an increased understanding of why the text messages sometimes functioned like ‘a kick in the pants’ and sometimes ran ‘like water off a duck’s back’. Timing in relation to precipitating events and motivation, and tailoring to prior knowledge, habits, and control beliefs thereby emerge as possible means to increase adoption.

### Findings in relation to other work

The first theme, ‘Blood pressure and lifestyle, how seriously to take it?’, revealed uncertainties regarding the relationship between lifestyle, hypertension, and disease. This is in line with deficiencies in patient knowledge of risk factors and effects of hypertension that have been reported previously [28]. The varying emphasis put on lifestyle during health care visits that some of our participants perceived also resonates with prior research, even if some progress can be discerned over time [29, 30]. However, a recent study reported poor communication about lifestyle as a barrier to hypertension management [31]. Thus, there seems to exist unmet needs for information on the linkage between different lifestyle factors, hypertension, and CVD as well as needs for specific lifestyle advice from health care providers. ‘Advice from a credible source’ was also one of the keys to success of a lifestyle text message intervention for CVD patients [15]. This corresponds to our factor ‘health care as the sender of lifestyle advice’ in the modified RAA, by which the text messages can affect perceived norms and by extension promote lifestyle change.

Text message interventions with lifestyle advice for patients with hypertension or CVD in different settings generally seem to be perceived as positive, useful, and tolerable [13–16, 32, 33]. This corresponds to our category ‘A useful and rational reminder’. Personalization and context-specific advice have been put forth as a means to increase effectiveness but have not been discussed in-depth [15, 31, 32]. A beneficial impact of the personal contact that some of our participants proposed has been indicated [15], but the absence of effects of two-way as compared to one-way text messages has also been reported [34]. However, one may draw parallels between the one-way communication through text messages and the term compliance as in following the physician’s prescriptions, and the newer and more patient-centered concept of concordance where cooperation between patient and professional is stressed, which would favor an intervention that included two-way communication [35]. Regarding the need for *more ‘eureka moments’,* i.e., more new advice or eye-opening suggestions, parallels are harder to find in prior research. A possible explanation is that our primary care, high-income setting involved patients with less serious conditions [9, 15, 16] and/or more knowledge [13, 14, 32] than populations of other studies, resulting in a need for stronger stimuli to change – which participants were also capable of expressing.

As lack of adoption seems to have been sparsely discussed in prior research on text message interventions [13–15, 32] our category ‘Like water off a duck’s back’ may add some new perspectives. Summing up the category, to increase adoption, it seems important to include information that is new to the participant, to not provoke feelings of guilt or illness, and to provide advice that is tailored enough to be applied by the participant. Advice contrary to strong control beliefs should be avoided, while advice that builds on the participants’ knowledge and existing habits should be prioritized. Thus, this category connects back to the suggestions of personalization and context-specific advice [15, 31, 32].

Our adoption category ‘A push to change’ primarily covers specific experiences and resulting lifestyle changes of the intervention, which agree with the overall findings of other qualitative studies [13, 15] and RCTs [12, 32]. Considering our findings more in detail, the synergistic effects of delivering the intervention in association with a precipitating event, including CVD, have been pointed out previously [13, 15]. The feeling of support, ‘someone is with me’, expressed by a few of our participants also echoes in some other interventions [13, 15, 32].

Regarding theoretical framework, prior research based on the TPB has primarily used instrumental (utilitarian) attitudes. However, the RAA was recently applied by La Babera and Ajzen (the originator of the theory) [27] to study intention to perform the WHO-recommended amount of physical activity. It was then found that experiential (affective) attitudes were better predictors of motivation to engage in physical activity [27]. In the context of our results, this may motivate an increased focus on making lifestyle change feel easier, for example through tailored suggestions of how to apply the advice.

Finally, the development process of seemingly simple text message interventions for CVD prevention can be laborious, and the question has been raised regarding whether interventions can be adapted between settings without the full developmental work being repeated [36]. In relation to other work, our analysis indicates that this may be feasible as several themes and needs are similar regardless of setting. However, the possibility to tailor advice to individual background factors may be a way to increase effectiveness in different settings.

### Strengths and limitations

We consider the use of an established theoretical framework for understanding and predicting behavior, the RAA, to be a strength of this study. The choice of the RAA was further motivated by the preceding framework of the TPB being used in another sub-study of PUSHME RCT, thus facilitating comparison and further development of results. We also used an established analytical framework that we perceived as adequate to systematize and interpret the material. The stepwise analysis used in systematic text condensation includes significant restructuring of the material during each analytical round, thus giving all interviews full weight on the results while keeping the material manageable to facilitate overview as well as focus [23, 24]. Furthermore, observer triangulation was repeatedly performed during the analysis to include viewpoints from all five researchers, which increases credibility.

All authors work as physicians at primary health care centers, with possible implications on their reflexivity as they have extensive preunderstanding of the study topic. HG conducted all interviews and coordinated the qualitative analysis, but did not take part in the RCT, which may have served to decrease researcher bias. The participants were informed that HG was a physician, which may have affected viewpoints that were expressed regarding subjects such as medication.

The method of data collection, telephone interviews, has advantages and disadvantages. Body language is lost, which can lead to poorer communication. However, inclusion of participants becomes easier. This was important as the study covered several regions and included participants of working age as well as risk groups for COVID-19. Perceived levels of trustfulness varied between interviews, but it was generally possible to elicit personal and often ample information through simple verbal cues and encouragement. The semi-structured interview guide was used as a memory aid, countering the risk for the interviews to be excessively controlled by the researcher resulting in poor material while still facilitating for each participant to convey their views regarding major questions of the study.

Regarding transferability, we achieved variability in participant characteristics including sex, age, location, education, lifestyle habits, blood pressure, and effects of the intervention. Viewpoints expressed, and personalities, also varied. This variation, in conjunction with several viewpoints finding echoes in prior research, provides that results may be transferable to text message interventions for cardiovascular prevention in other contexts. However, we consider results less transferable to other modes of digital interventions and to other conditions as participants were interviewed specifically about text messages, lifestyle, and hypertension.

### Clinical implications and further research

Our findings indicate that text messages with lifestyle advice for patients with hypertension are tolerated and often appreciated as an addition to the usual management of hypertension in primary care in a high-income country. From this viewpoint, the intervention could be used in clinical practice to contribute to the prevention of CVD. Timing in relation to a diagnosis of hypertension or other motivational factors, and tailoring to prior knowledge, habits, and limitations could increase effectiveness. Constructing text messages with the aim of eliciting some ‘eureka moments’ may also be evaluated.
